# Three-Dimensional Culture Promotes the Differentiation of Human Dental Pulp Mesenchymal Stem Cells Into Insulin-Producing Cells for Improving the Diabetes Therapy

**DOI:** 10.3389/fphar.2019.01576

**Published:** 2020-01-24

**Authors:** Bingbing Xu, Daoyang Fan, Yunshan Zhao, Jing Li, Zhendong Wang, Jianhua Wang, Xiuwei Wang, Zhen Guan, Bo Niu

**Affiliations:** ^1^ Department of Translational Medicine, Capital Institute of Pediatrics, Beijing, China; ^2^ Graduate School of Peking Union Medical College, Beijing, China; ^3^ Knee Surgery Department of the Institute of Sports Medicine, Beijing Key Laboratory of Sports Injuries, Peking University Third Hospital, Beijing, China; ^4^ Institute of General Surgery, Chinese PLA General Hospital, Beijing, China; ^5^ Laboratory of Translational Medicine, Chinese PLA General Hospital, Beijing, China; ^6^ Department of Biochemistry and Molecular Biology, School of Basic Medical Sciences, Shanxi Medical University, Taiyuan, China

**Keywords:** diabetes mellitus, regenerative medicine, human dental pulp stem cells, insulin-producing cells, Matrigel biomaterial

## Abstract

**Introduction:**

Diabetes is a metabolic disease with a high incidence and serious harm to human health. Islet β-cell function defects can occur in the late stage of type 1 diabetes and type 2 diabetes. Studies have shown that stem cell is a promising new approach in bioengineering regenerative medicine. In the study of stem cell differentiation, three-dimensional (3D) cell culture is more capable of mimicking the microenvironment of cell growth *in vivo* than two-dimensional (2D) cell culture. The natural contact between cells and cells, and cells and extracellular matrix can regulate the development process and promote the formation of the artificial regenerative organs and organization. Type IV, VI collagen and laminin are the most abundant extracellular matrix components in islets. Matrigel, a basement membrane matrix biomaterial rich in laminin and collagen IV.

**Materials and Methods:**

We used Matrigel biomaterial to physically embed human dental pulp stem cells (hDPSCs) to provide vector and 3D culture conditions for cells, and we explored and compared the preparation methods and preliminary mechanisms of differentiation of hDPSCs into insulin-producing cells (IPCs) under 2D or 3D culture conditions.We first designed and screened the strategy by mimicking the critical events of pancreatogenesis *in vivo*, and succeeded in establishing a new method for obtaining IPCs from hDPSCs. Activin A, Noggin, and small molecule compounds were used to synergistically induce hDPSCs to differentiate into definitive endoderm-like cells, pancreatic progenitor like cells and IPCs step by step under 2D culture conditions. Then, we used Matrigel to simulate the microenvironment *in vivo*, induced hDPSCs to differentiate into IPCs in Matrigel, evaluated and compared the efficiency between 2D and 3D culture conditions.

**Results:**

The results showed that the synergistic combination of growth factors and small molecule compounds and 3D culture promoted the differentiation of hDPSCs into IPCs, significantly enhancing the release of insulin and C-peptide from IPCs.

**Discussion:**

Significant support is provided for obtaining a large number of functional IPCs for disease modeling and final cell therapy in regenerative medicine.

## Introduction

Diabetes is a global public health problem that seriously threatens the health of more than 400 million people, and the prevalence has been increasing (Stokes and Preston, 2017). At present, the main treatments for diabetes include diet control, regular blood glucose testing, oral hypoglycemic chemotherapy and insulin supplementation. However, such treatment methods can not reconstruct the physiological blood sugar regulation function of the body, and can not avoid the occurrence of severe hypoglycemia and long-term complications (Nathan, 1993). Another treatment method is to replace endogenous β cells by islet transplantation. Islet transplantation can reduce the dependence of patients with exogenous insulin, and effectively control blood sugar levels and reduce the occurrence of complications by increasing the number of islet cells with endocrine function to treat or even cure diabetes (Shapiro et al., 2000; Bellin et al., 2012; Hering et al., 2016). Although islet transplantation has been successfully used in clinical practice, the limited supply of islets and the side effects of immunosuppressive therapy have largely hampered the widespread use of this therapy (Porat and Dor, 2007), prompting us to search for alternative sources of islet cells.

Human mesenchymal stem cells (MSCs)-derived β cells are the most promising alternative source of cells for use in diabetic cell replacement therapy and other applications, such as mimicking disease and studying pancreas development (Millman and Pagliuca, 2017). Studies have shown that liver stem cells (Yang et al., 2002), umbilical cord blood (Koblas et al., 2009), bone marrow (Chen et al., 2004), adipose tissue-derived MSCs (Li et al., 2013) have the potential to differentiate into insulin-producing cells (IPCs). However, the scarcity of tissue sources, the highly invasive procedures for acquiring tissues, and the considerable risk of morbidity at the donor site limit their use. The human dental pulp stem cells (hDPSCs) are rich in source, easy to obtain, non-invasively separated, donors have no discomfort, and there is no ethical controversy in research and development applications, which is undoubtedly an attractive source of MSCs.

Recent studies have shown that some small molecule compounds can regulate the different developmental stages of islet beta cells by regulating embryonic developmental pathways. Lithium chloride (LiCl), in place of recombinant Wnt 3a protein, activates typical Wnt signaling and promotes differentiation of ESCs into DE cells (Li et al., 2011). Retinoic acid can promote the expression of pancreatic endocrine progenitor cells and further differentiate into islet β cells (Oström et al., 2008; Cai et al., 2010); A83-01 is a selective inhibitor of transforming growth factor-β (TGF-β) type 1 receptor ALK5 kinase, which blocks phosphorylation of Smad2, inhibits TGF-β-induced epithelial–mesenchymal transition and promotes the expression of pancreatic progenitor cells; LDE225 is an FDA-approved drug for cancer patients and is an inhibitor of the Hedgehog pathway (Rimkus et al., 2016), which replaces FGF2 protein to exclude Hedgehog signaling and promotes pancreatic lineage formation (Kim and Hebrok, 2001; Li et al., 2014); 2-Phospho-L-ascorbic acid trisodium salt (pVc) promotes differentiation and maturation of ESCs and fibroblasts to insulin-secreting cells (Li et al., 2014); SB203580 is a p38 mitogen-activated protein kinase inhibitor that improves insulin-secreting cell function by promoting differentiation and maturation of insulin-secreting cells (Li et al., 2014; Wei et al., 2018).

Studies have reported different sources of cells such as mouse ESCs (Li et al., 2011), human MSCs (Wang et al., 2010), Human induced pluripotent stem cells (hiPS) (Kunisada et al., 2012) and mouse fibroblasts (Li et al., 2014), all can be induced to differentiate into IPCs by small molecule compounds. But so far hDPSCs have not been reported, so we designed and screened the strategy by mimicking the critical events of pancreatogenesis *in vivo*. Activin A, Noggin and small molecule compounds were used to synergistically induce hDPSCs to differentiate into definitive endoderm-like cells, pancreatic progenitorlike cells and IPCs step by step under 2D culture conditions. The efficiency of induction was assessed by methods such as reverse transcription quantitative polymerase chain reaction, immunocytochemical staining, and insulin secretion function assays.

Three-dimensional (3D) cell culture technology refers to the use of different methods and materials to simulate the growth environment *in vivo*, so that cells can grow and migrate in a 3D space, and promote cell differentiation, which is conducive to its function (Wang et al., 2019). A series of evidence suggests that the extracellular matrix (ECM) provides a spatial and temporally controlled environmental support for stem cell differentiation and tissue maturation, responsible for structural and biochemical support, regulates molecular signaling and tissue repair in many organ structures, including the pancreas. In islets, type IV and VI collagen and laminin are the most abundant molecules in ECM compared to other ECM molecules. Some ECM molecules are involved in the survival, function, and insulin production of beta cells, while other ECM molecules regulate the sensitivity of islet cells to cytokines (Llacua et al., 2018).

In the study of stem cell differentiation, 3D cell culture is more capable of mimicking the microenvironment of cell growth *in vivo* than 2D cell culture. The natural contact between cells and cells, between cells and ECM can regulate the development process and promote the formation of artificial organs and Organizational (Zhang et al., 2019a; Zhang et al., 2019b); 3D cell culture can perfectly reproduce the process of embryo development *in vivo*, and facilitate the further study of its molecular mechanism (Gelain et al., 2006; Albrecht et al., 2006). In the 3D microenvironment, human embryonic stem cells are more efficiently differentiated into pancreatic endocrine cells, more pronounced response to glucose (Wang et al., 2017).

The Matrigel biomaterial basement membrane matrix is derived from mouse sarcoma, the main component of which is laminin, followed by collagen IV, heparan sulfate proteoglycan and nestin (Kleinman et al., 1986; Wang et al., 2017). At room temperature (RT), Matrigel polymerizes to form a biologically active 3D matrix that mimics the structure, composition, physical properties and functions of the cell basement membrane *in vivo*, facilitating cell culture and differentiation *in vitro*. Matrigel can be used for the study of cell morphology, biochemical function, migration, infection and gene expression, and is suitable as a matrix material for the differentiation of hDPSCs into IPCs. By simulating the *in vivo* microenvironment, we first cultured hDPSCs in Matrigel rich in laminin and collagen IV to induce the differentiation of hDPSCs into insulin-secreting cells, and we compared the difference between 2D induction and 3D induction.

Our protocol can efficiently produce functional IPCs under both 2D and 3D culture conditions. Our results highlight the synergistic approach between growth factors and small molecule compounds and the important role of Matrigel in inducing hDPSCs to differentiate into IPCs. Significant support is provided for obtaining a large number of functional IPCs for disease modeling and final cell therapy in regenerative medicine.

## Materials and Methods

### Materials

Dulbecco’s modified Eagle’s medium/nutrient mixture F-12 (DMEM-F12), penicillin/streptomycin, and fetal bovine serum (FBS) were purchased from Gibco. Anti-human CD34-PE, CD44-FITC, CD45-FITC, CD73-PE, CD90-FITC, and HLA-DR-FITC were obtained from BD Biosciences. Adipogenic induction medium and osteogenic induction medium Cyagen. Primary antibodies (Sox17, Cxcr4, Pdx1, and Glucagon) and fluorescent secondary antibodies were purchased from Abcam. Primary antibodies (Nkx6.1, Insulin, Somatostatin) were purchased from CST. A83-01 and SB203580 were purchased from Tocris. LDE225 were obtained from Selleck. Activin, Noggin human and other small molecule compounds were purchased from Sigma. Matrigel were purchased from Corning.

### Isolation and Culture of Human Dental Pulp Stem Cells

Sound intact deciduous tooth were extracted from 20 donors (ages 8–12-year old of children) who were undergoing a continuous extraction for occlusion treatment. Written informed consents were obtained from donors and guardians. The experiments involving human tissue were approved by Capital Institute of Pediatrics and were all carried out in accordance with the ethical standards of the local ethical committee.

The deciduous teeth were washed two to three times with physiological saline. The teeth crown was fixed with hemostatic forceps and the teeth root was crushed with a rongeur to expose the pulp. The pulp tissue was minced into small fragments before digestion in a solution of 0.05% *(w/v)* collagenase P for 30 min at 37°C, 180 rpm in a constant temperature shaker, and then filtered through a 100 μm nylon cell strainer. The next procedures, culture conditions and media were applied as described for human endometrial stem cells.

### Flow Cytometry Analysis

For phenotypic identification of the hDPSCs at P4, cells (1 × 10^6^) were digested with 0.25% (w/v) trypsin, washed twice with phosphate-buffered saline (PBS) and divided into aliquots. The cells were centrifuged, resuspended and stained with the following antibodies for 15 min at RT: anti-human CD34-PE, CD44-FITC, CD45-FITC, CD73-PE, CD90-FITC, and HLA-DR-FITC (BD Biosciences, USA). After washing, the cells were resuspended and then analyzed using flow cytometry instrument (FC500; Beckman Coulter, USA).

### *In Vitro* Multilineage Differentiation Assay for Human Dental Pulp Stem Cells

hDPSCs at P4 were differentiated into adipocytes and osteoblasts as follows *in vitro*. For adipogenic and osteogenic differentiation, hDPSCs were reseeded in the standard culture medium into 6-well culture plates at 2 × 10^4^ cells per well. The cells were incubated until they are 100% confluent or post-confluent. hDPSCs were exposed to adipogenic induction medium for 28 days. hDPSCs were exposed to osteogenic induction medium (Cyagen) for 28 days. The medium was replaced with fresh induction medium every 3 days. After 7 or 28 days, cells were fixed with 4% (w/v) paraformaldehyde solution. To assess adipogenic differentiation, cells were stained with oil red O working solution. To assess osteogenic differentiation, cells were stained with alizarin red working solution. Control cells were cultured in standard culture medium over the same period of time. The stained plates were visualized under light microscope (Leica DM IL LED Fluo, Leica Microsystems Inc, Germany) and captured images using Leica Application Suite Version 4.5.0 software.

### *In Vitro* Differentiation Assay of Human Dental Pulp Stem Cells Into Insulin-Producing Cells

Differentiation of hDPSCs into IPCs was carried out in 3 stages by Method 1 (M 1). At stage 1, for differentiation into DELCs, hDPSCs was treated with the DMEM/F12 medium supplemented with 1.92 nM Activin A, 1 mM LiCl, 180 ng/ml Noggin, 280 μM pVc, β-ME, and 1% FBS for 6 days. At stage 2, for differentiation into PPLCs, the cells were cultured in induction medium comprising DMEM/F12 and 2 μM retinoic acid, 1 μM A83-01, 2 μM LDE225, 280 μM pVc, and 1% FBS for 1 day on day 6. After 1 day, the cells were treated with the DMEM/F12 medium containing 1 μM A83-01, 2 μM LDE225, 280 μM pVc, and 1% FBS for 3 days. At stage 3, for differentiation into IPCs, the cells were cultured in induction medium comprising DMEM/F12, 5 μM SB, 280 μM pVc, ITS, 1 mM nicotinamide, 1× non-essential amino acids, and 1% FBS for 10 days. The study compared the new method 1 (M 1) with method 2(M 2); The M 2 came from Govindasamy et al. (Govindasamy et al., 2011), slightly modified as M 2. Undifferentiated hDPSCs with the same culture days were used as controls. Fresh induced differentiation medium changed every 2 days.

### RNA Isolation and RT-qPCR Analysis

Cultured hDPSCs in P4 were collected and total RNA were extracted with Trizol Reagent (Invitrogen). cDNA was synthesized from RNA using All-In-One RT MasterMix Kit (abmGood, Canada). qPCR was performed using EvaGreen qPCR MasterMix Kit (abmGood, Canada). Thermal cycling conditions were 95°C for 10 min and 40 cycles of 95°C for 15 s, 60°C for 60 s. The sequences of primers were listed in **Table 1**. The gene expression level of glyceraldehyde 3-phosphate dehydrogenase (GAPDH) was served as an internal reference.

**Table 1:** The sequences of primers.

Primers	Sequences
Sox17-Forward	5’-GGCGCAGCAGAATCCAGA-3’
Sox17-Reverse	5’-CCACGACTTGCCCAGCAT-3’
Foxa2-Forward	5’-AAGACCTACAGGCGCAGCTA-3’
Foxa2-Reverse	5’-CCTTCAGGAAACAGTCGTTGA-3’
Cxcr4-Forward	5’-AACTGAGAAGCATGACGGACAAGTAC-3’
Cxcr4-Reverse	5’-GCTGTAGAGGTTGACTGTGTAGATGAC-3’
Pdx1-Forward	5’-TGATACTGGATTGGCGTTGT-3’
Pdx1-Reverse	5’-GAATGGCTTTATGGCAGATTA-3’
Nkx6.1-Forward	5’-GGCCTGTACCCCTCATCAAG-3’
Nkx6.1-Reverse	5’-CCGGAAAAAGTGGGTCTCGT-3’
Neurod1- Forward	5’-GACGACCTCGAAGCCATGAACG-3’
Neurod1- Reverse	5’-CCTCCTCTTCCTCTTCTTCCTCCTC-3’
Ngn3- Forward	5’-GGCTGTGGGTGCTAAGGGTA-3’
Ngn3- Reverse	5’-CAGGGAGAAGCAGAAGGAACAA-3’
Pax4-Forward	5’-GTATGGCTTGGAATGAGGCAGGAG-3’
Pax4-Reverse	5’-GCAATCACAGGAAGGAGGAAGGAG-3’
Insulin-Forward	5’-CAGCCGCAGCCTTTGTGA-3’
Insulin-Reverse	5’-GTGTAGAAGAAGCCTCGTTCC-3’
Glucagon-Forward	5’-AAGAGGTCGCCATTGTTG-3’
Glucagon-Reverse	5’-TAGCAGGTGATGTTGTGAAG-3’
Gapdh-Forward	5’-CAGGAGGCATTGCTGATGAT-3’
Gapdh-Reverse	5’-GAAGGCTGGGGCTCATTT-3’

### Immunocytochemistry Analysis

Undifferentiated DPSCs or IPCs were fixed for 30 min in 4% paraformaldehyde, treated with 0.1% Triton-PBS for optimal penetration of cell membranes, and incubated at RT for 30 min, treated with blocking solution (5% BSA) RT for 30 min. And the primary antibodies were incubated overnight at 4°C, washed with PBST for three times, and then incubated with secondary antibodies at 37°C for 40 min. Slides were counterstained with 4′,6′-diamidino-2-phenylindole dihydrochloride (DAPI) for 5 min. Fluorescent images were captured by Leica TCS SP8 microscope. The dilutions of antibodies are according to the instructions.

### Insulin and C-Peptide Release Assay

Cells were washed five times and pre-incubated for 90 min at 37°C in the freshly prepared Krebs’ Ringer bicarbonate 4-(2-hydroxyethyl)-1-piperazineethanesulfonic acid (HEPES) buffer (KRBH; 4.7 mM KCl, 118 mM NaCl, 25 mM NaHCO_3_, 1.1 mM KH_2_PO_4_, 2.5 mM MgSO_4_, 3.4 mM CaCl_2_, 10 mM HEPES, and 2 mg/ml BSA, pH 7.4) containing 2.8 mM glucose. The supernatant was then collected and replaced with Krebs’ Ringer bicarbonate HEPES buffer supplemented with 16.7 mM D-glucose at 37°C for another 90 min. The Supernatant was also collected to detect the release of insulin and C-peptide by Roche Cobas 6000 automatic electrochemiluminescence analyzer. The multiple stimulation of each culture was calculated by dividing the concentration of insulin or C-peptide in the stimulation supernatant by the concentration of insulin or C-peptide in the basal supernatant.

### Statistical Analysis

Parametric data are presented as the means ± standard deviation (SD). GraphPad Prism version 5.0 (California, USA) was used for the statistical analyses. All statistical comparisons between two groups were performed using the two-sided, nonpaired *t*-test. Differences were considered significant at p < 0.05.

## Results and Discussion

### Morphology and Multilineage Differentiation Potential

**Figure 1:**
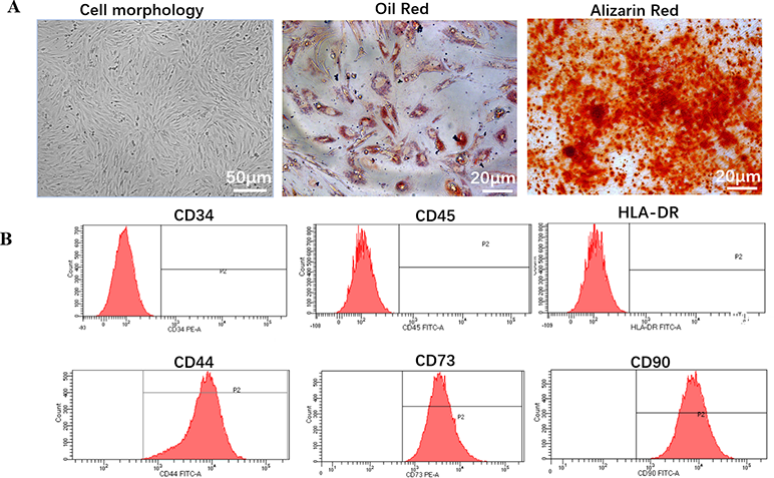
Biology characteristic of hDPSCs. **(A)** Morphology and multilineage differentiation of hDPSCs. All MSCs exhibited a similar fibroblast-like, spindle-shaped morphology (left panel, scale bar: 50 µm) and were induced to differentiate toward adipogenic lineage verified by Oil Red O (middle panel, scale bar: 20 µm) and osteogenic lineage verified by Alizarin Red (right panel, scale bar: 20 µm). Shown is one representative of 3 independent experiments. **(B)** Flow cytometric analysis of the expression of surface markers on hDPSCs. hDPSCs showed high expression of MSC-specific surface markers (CD44, CD73, and CD90), and low expression of leucocyte marker (CD45), hematopoietic cell marker (CD34), or monocyte/macrophage marker (HLA-DR). Shown is one representative of 3 independent experiments.

hDPSCs exhibited typical MSC morphology, and shared the similar fibroblast-like, spindle shape and expressed high capacity to adhere to plastic culture flasks (**Figure 1A**).

To investigate the differentiation potential of the hDPSCs, the cells were directed toward the osteogenic and adipogenic lineages at P4 (**Figure 1A**). By direct observation under the microscope, lipid droplets began to appear in hDPSCs on about day 21. After 28 days of induction with adipogenic induction medium, adipogenic differentiation was evaluated by cytoplasmic lipid droplets and Oil Red O staining. Only very small lipid granules were detected in hDPSCs on day 28. After 28 days of induction with osteogenic induction medium, osteogenic differentiation was verified by calcium deposition and alizarin red staining. hDPSCs possessed an osteogenic phenotype and also differentiated into osteocytes. Therefore, we suggested that hDPSCs can differentiate into two abovementioned lineages.

### Immunophenotype

We investigated hDPSCs immunophenotype at P4 using flow cytometry instrument. A series of classical MSCs phenotypic markers as defined by the International Society for Cellular Therapy criteria were examined, including CD34, CD44, CD45, CD73, CD90, and HLA-DR. hDPSCs showed high expression of MSC-specific surface markers (CD44, CD73, and CD90), and low expression of leucocyte marker (CD45), hematopoietic cell marker (CD34) and monocyte/macrophage marker (HLA-DR) (**Figure 1B**).

### Differentiation of Dental Pulp Stem Cells Into Insulin-Producing Cells in 2-Dimensional Cultures

**Figure 2:**
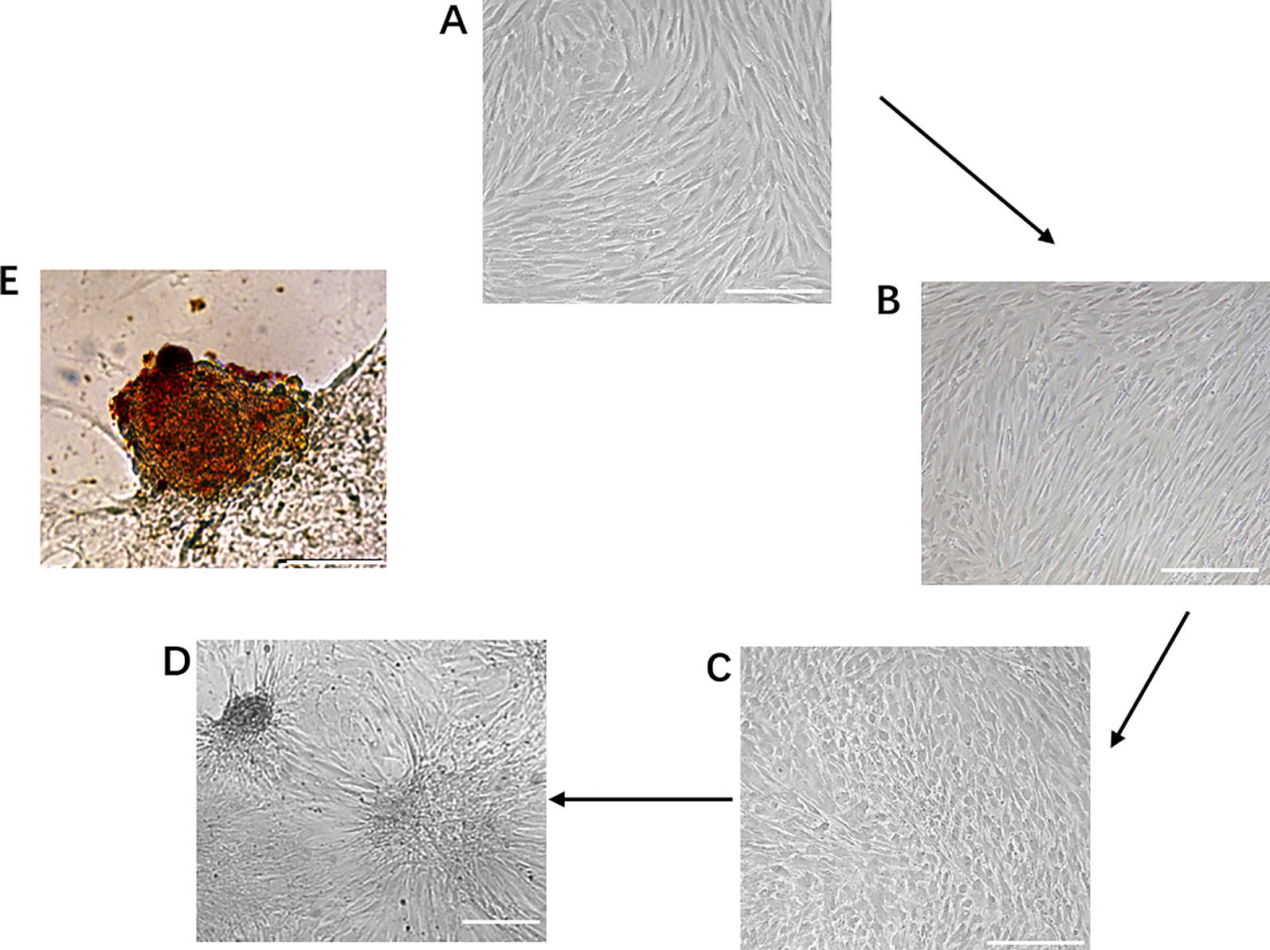
Generation of IPCs from hDPSCs. **(A**–**D)** Phenotypic changes in hDPSCs during the 19-day differentiation procedure. Scale bars: 50 μm. **(E)** Dithizone staining. Dithizone staining showed that the day 19 IPCs were scarlet. Dithizone can specifically chelate zinc ions, indicating that the cytoplasm of the day 19 IPCs is rich in zinc ions specific to islet β cells. Scale bars: 25 μm.

During the first phase of induction, human dental pulp mesenchymal stem cells were transformed from the original fibroblast-like morphology (**Figure 2A**) to a sparse long spindle shape (**Figure 2B**). During the second phase of induction, the cells gradually changed from long fusiform to short fusiform, elliptical and round, and voids appeared between cells in the local area, and the cells had a tendency to aggregate (**Figure 2C**). During the third phase of induction, the tendency of cell aggregation is more obvious, and cell clusters gradually form between cells, and the number of cell clusters gradually increases (**Figure 2D**). Dithizone staining showed that the cells after induction were scarlet and the undifferentiated cells cannot be stained. Dithizone can specifically chelate zinc ions, indicating that the cytoplasm of cells is rich in zinc ions specific to islet β cells (**Figure 2E**).

**Figure 3:**
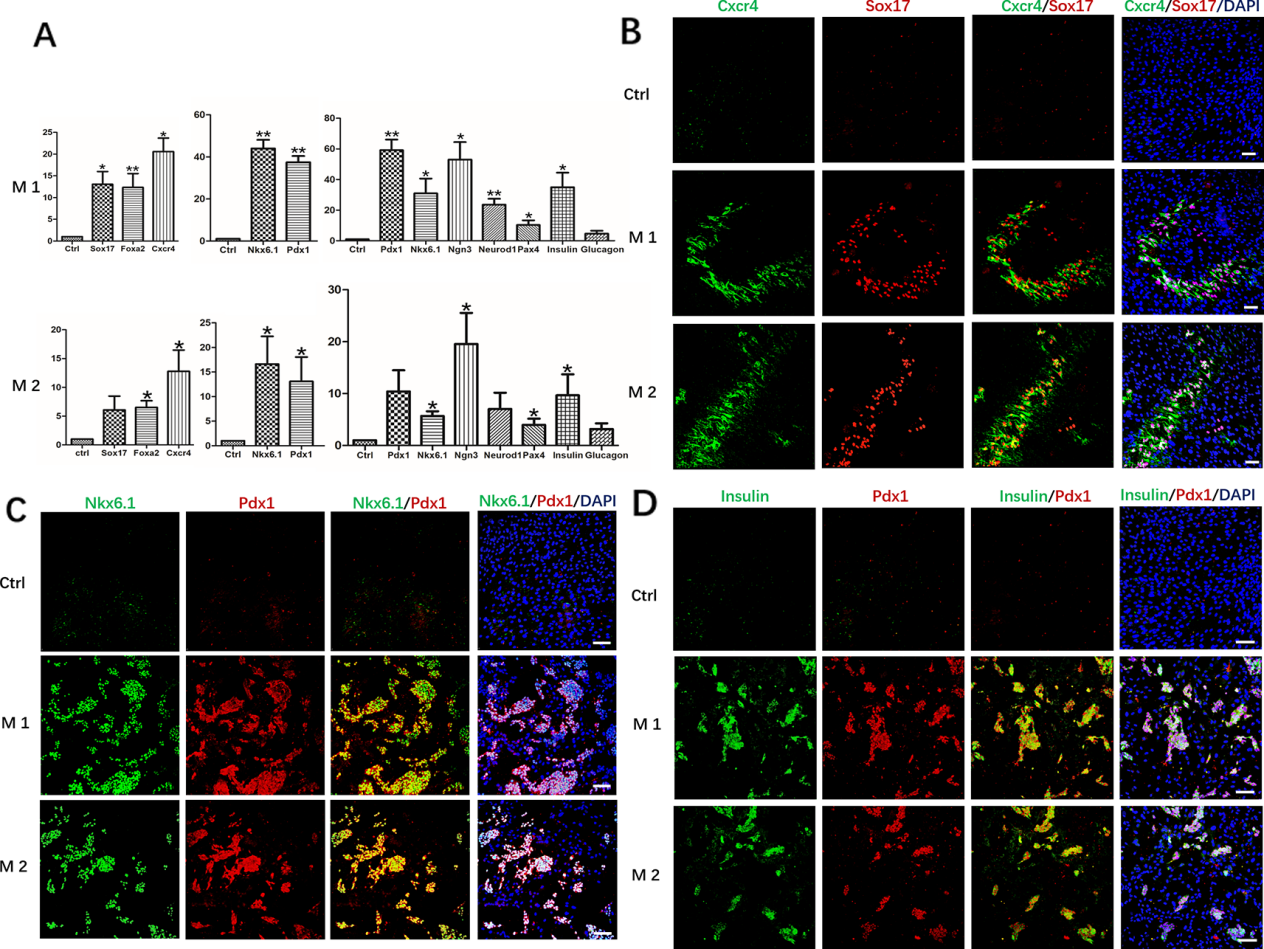
Expression of marker genes at three differentiation stages of cells. **(A)** RT-PCR was performed to analyze the status of marker genes during the differentiation process. Results were the average of three independent experiments. *p < 0.05, **p < 0.01. **(B)** Immunostaining of definitive endoderm markers Cxcr4 and Sox17 on day 5. **(C)** Immunostaining of definitive endoderm markers pancreatic progenitor markers Pdx1 and Nkx6.1 on day 9. **(D)** Immunostaining of pancreatic cell markers Pdx1 and Insulin on day 19. Undifferentiated DPSCs were used as controls. Scale bars: 100 μm.

After induction at different stages, undifferentiated hDPSCs with the same culture days were used as controls, RT-qPCR analysis revealed mRNA expression levels of the definitive endoderm marker genes FOXA2, CXCR4, and SOX17, pancreatic progenitor marker genes Pdx1 and Nkx6.1, pancreatic cell marker genes PDX1, NKX6.1, NGN3, NEUROD1, PAX4, Insulin and Glucagon, all were maintained at different higher levels in Method 1 than that of Method 2 (**Figure 3A**). The results were further confirmed by immunofluorescence staining. The protein level identified by immunofluorescence was consistent with the level of RT-qPCR levels. After identification and analysis by immunofluorescence observation and software image-ProPlus6.0, we found that the proportion of Cxcr4+/Sox17+cells was approximately 26% after the first phase of induction, that of Method 2 was 20% (**Figure 3B**). About 35% of cells were Pdx1+/Nkx6.1+cells after the second phase of induction, which was also higher than that of Method 2(21%) (**Figure 3C**). After induction in the third stage, the proportion of Insulin+/PDX1+cells was approximately 36%. However, only 15% of cells were Pdx1+/Nkx6.1+cells in Method 2 (**Figure 3D**).

**Figure 4:**
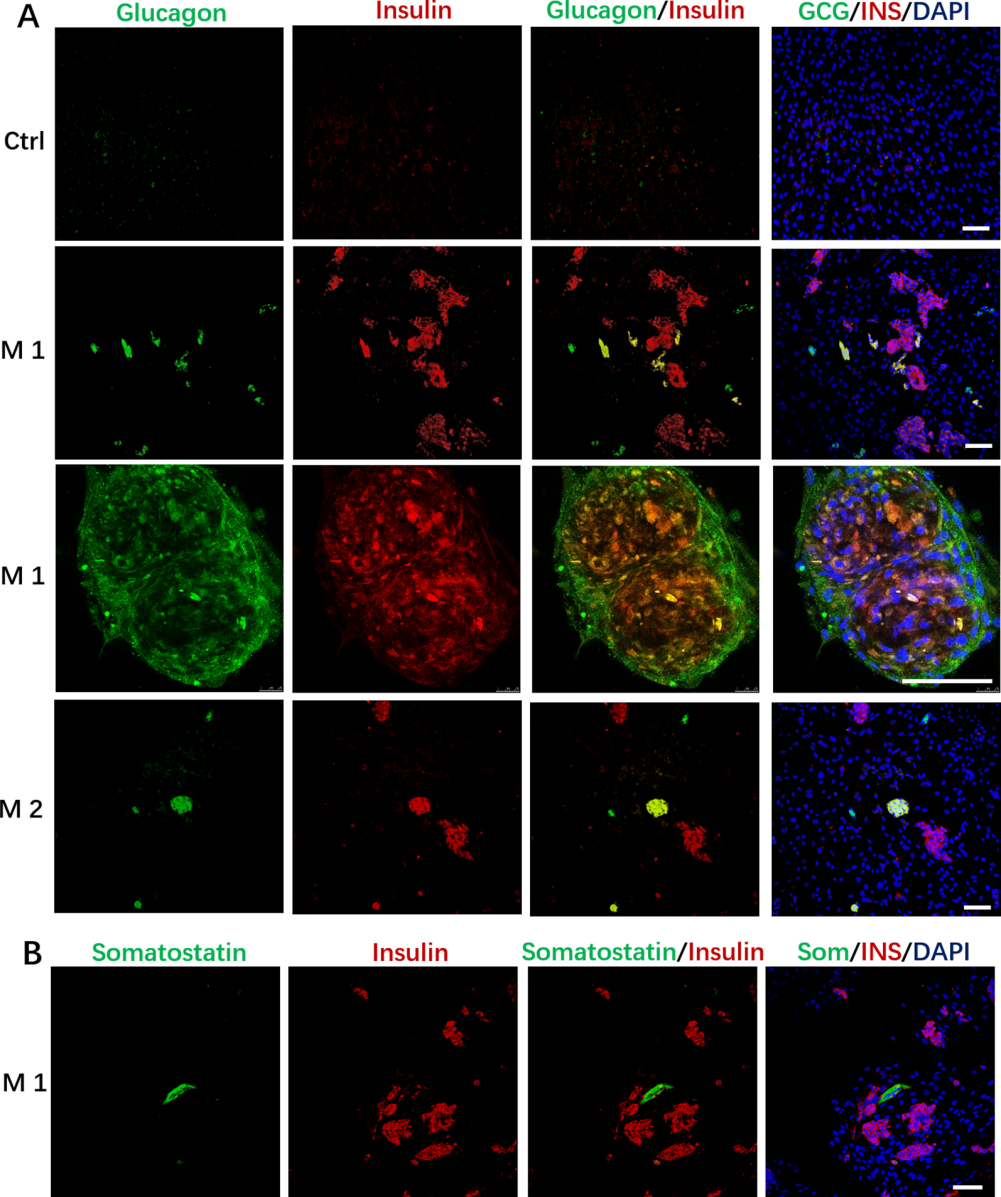
Immunostaining of islet markers on day 19. **(A)** Immunostaining of islet cell markers Glucagon and Insulin on day 19. Cells in M 1 forming a cell aggregate like islet-like cells in the differentiated culture were colocalized with Insulin and Glucagon expression, and cells in M 1 at the edge of the cell aggregate were Glucagon positive. **(B)** Immunostaining of islet cell markers Somatostatin and Insulin on day 19 in M 1. Scale bars: 100 μm. Undifferentiated DPSCs were used as controls.

In addition to the discovery of Insulin+/PDX1+cells, we also found that the induced cells contained some Glucagon+ cells, and a small number of cells simultaneously expressed insulin and glucagon in the M 1 and M2 (**Figure 4A**); In addition, we observed that cells in M 1 forming a cell aggregate like islet-like cells in the differentiated culture were colocalized with Insulin and Glucagon expression, and cells in M 1 at the edge of the cell cluster were Glucagon positive (**Figure 4A**).These cells that simultaneously express insulin and glucagon are often described as immature or fetal-like endocrine cells. Finally, we also found that the cells induced by the M 1 contained very small amounts of Somatostatin-positive cells (**Figure 4B**).

### Differentiation of Dental Pulp Stem Cells Into IPCs in Three-Dimensional Culture

**Figure 5:**
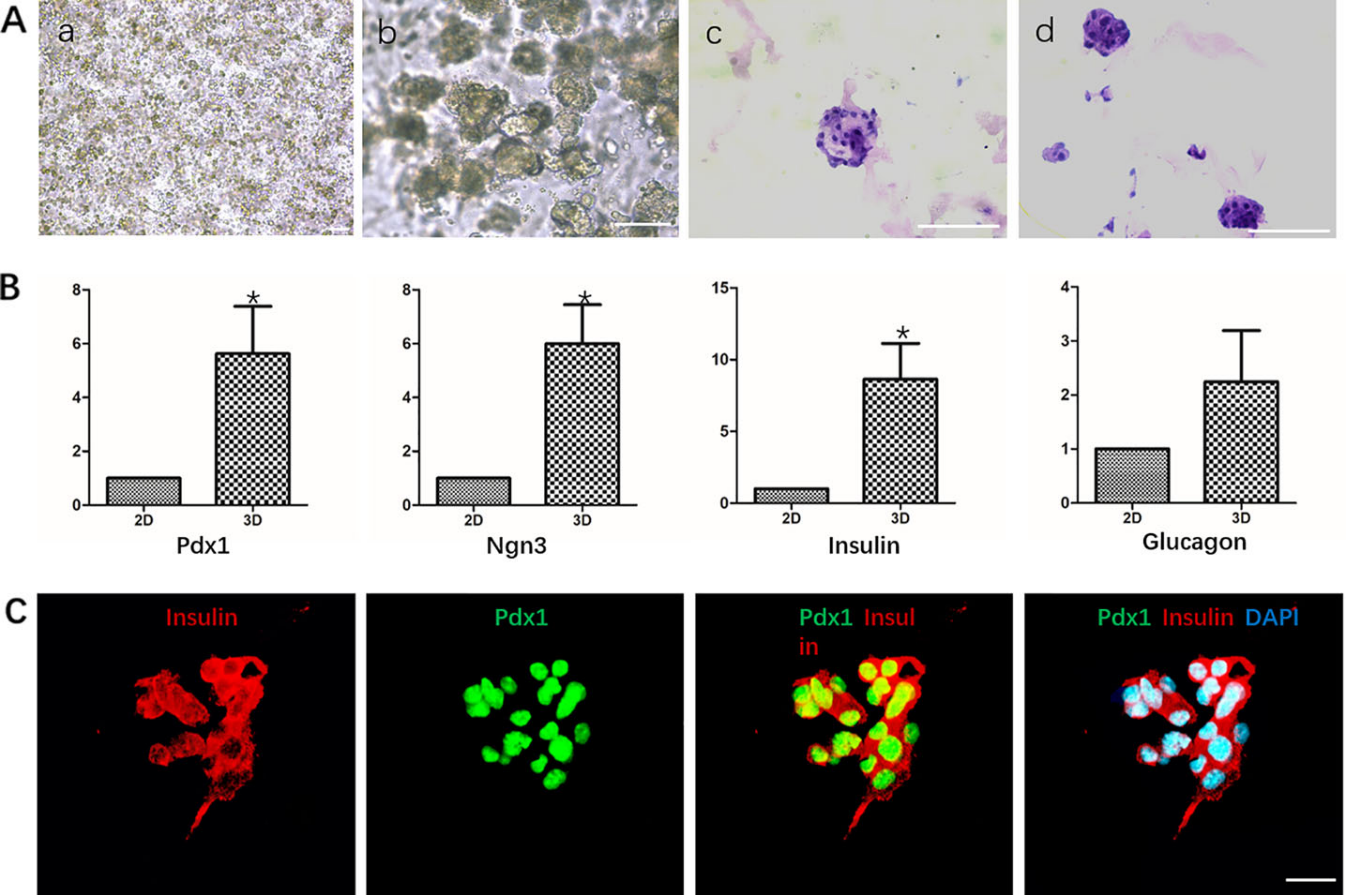
Differentiation of hDPSCs into IPCs in Matrigel. **(A)** Analysis of the growth morphology of hDPSCs in Matrigel. a. Matrigel physical embedded cells before induction; b. Growth status of cells in Matrigel after induction; c-d. HE staining analysis of different cell aggregates after induction. Scale bars: 20 μm. **(B)** Gene expression analysis Pdx1, Nkx6.1, Insulin and Glucagon by qPCR. Cells in 2D group were used as control. Results are the average of three independent experiments. *p < 0.05. **(C)** Immunostaining of pancreatic cell markers Pdx1 and Insulin on day 19. Scale bars: 20 μm.

We used Matrigel to physically embed hDPSCs to provide vector and three-dimensional culture conditions for cells. HE staining analysis showed that DPSCs grew in small globular cell clusters during the differentiation process, and the cells grew well (**Figure 5A**).

RT-qPCR analysis showed that the mRNA levels of islet cell marker genes PDX1, NGN3, and Insulin in the third induction stage of the 3D group were significantly higher than those in the 2D group, which was statistically significant. The mRNA level of Glucagon was also higher than that of the 2D group, but the difference was not significant (**Figure 5B**). Immunocytochemistry analysis showed that cells in the cell mass were highly expressed Pdx1 and Insulin (**Figure 5C**).

### Static Stimulation and Insulin/C-Peptide Content of Insulin-Producing Cells

**Figure 6:**
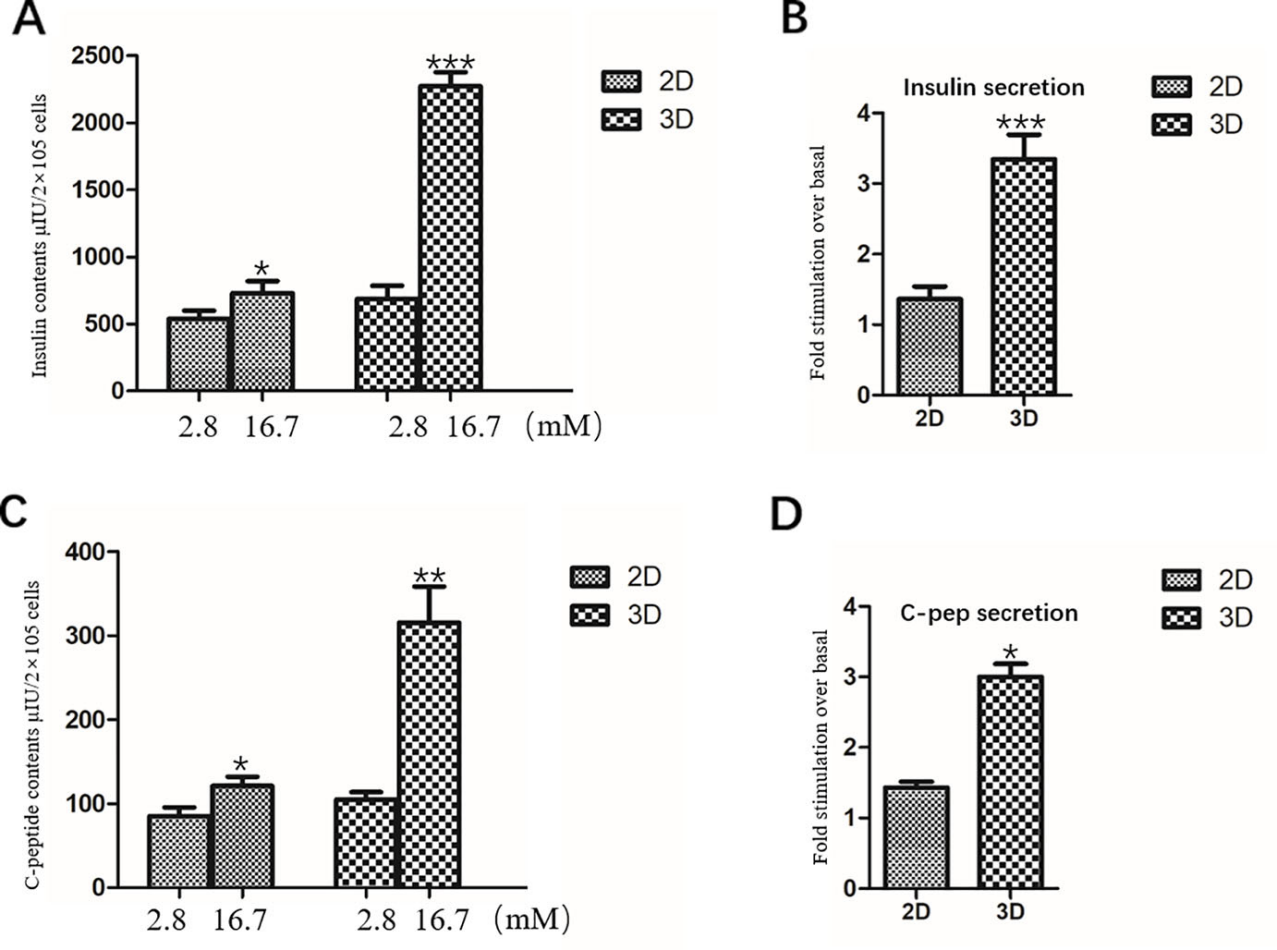
Insulin and C-peptide Release Assay in 2D and 3D group. **(A)** The total release insulin contents of IPCs when exposed to 2.8 and 16.7 mM glucose in the 2D group and 3D group. **(B)** Fold stimulation of insulin release over the respective basal condition for glucose in the 2D group and 3D group. **(C)** The total release C-peptide contents of IPCs when exposed to 2.8 and 16.7 mM glucose in the 2D group and 3D group. **(D)** Fold stimulation of C-peptide release over the respective basal condition for glucose in the 2D group and 3D group. Results are the average of three independent experiments. *p <0.05, **p <0.01, ***p <0.001.

IPCs in the 2D group were exposed to 2.8 mM glucose and 16.7 mM glucose, respectively. The total insulin and C-peptide release contents of IPCs when exposed to 2.8 mM glucose were 539.2 ± 60.68 μU/2×10^5^ cells (**Figure 6A**) and 84.82 ± 10.73 pmol/2×10^5^ cells (**Figure 6C**), respectively; When stimulated with 16.7mM glucose, the total insulin and C-peptide release contents were 729.6 ± 90.87 μU/2×10^5^ cells (**Figure 6A**) and 120.89 ± 10.84 pmol/2×10^5^ cells (**Figure 6C**), respectively, confirming their ability to respond to glucose *in vitro*.

The total insulin release contents and C-peptide release contents of IPCs when exposed to 16.7 mM glucose in the 3D group (2273.06 ± 104.4 μU/2×10^5^ cells, 315.26 ± 43.07 pmol/2×10^5^ cells) was significantly higher than that in the low concentration 2.8 mM glucose-stimulated group (686.16 ± 98.37 μU/2×10^5^ cells, 104.74 ± 8.95 pmol/2×10^5^ cells), which was statistically significant (**Figures 6A, C**). The total insulin release contents and C-peptide release contents of IPCs when exposed to 16.7 mM glucose in the 3D group was significantly higher than that in 2D group, which was statistically significant (**Figures 6A, C**).Fold stimulation of insulin and C-peptide release over the respective basal condition for glucose in the 3D group (3.35 ± 0.35, 3.00 ± 0.18) was significantly higher than that in 2D group (1.36 ± 0.18, 1.43 ± 0.08), which was statistically significant (**Figures 6B, D**). The results of insulin and C-peptide release assay further indicated that the IPCs in the 3D group were more sensitive to glucose response. The 3D culture conditions promoted insulin synthesis in IPCs, and the insulin release function was more mature.

## Conclusions

hDPSCs are a promising source of stem cells in tissue engineering therapy because they are less expensive to acquire and ease of harvest than the costly and invasive techniques required to isolate other mesenchymal stem cells (Han et al., 2019). DPSCs usually come from deciduous teeth, third molars, extra or orthodontic teeth, without adversely affecting the health of permanent dental pulp tissue (Shoi et al., 2014; Ledesma-Martínez et al., 2016). The morphology of hDPSCs showed typical morphology of MSCs, which was relatively uniform in morphology, similar to the fusiform shape of fibroblasts, and showed high adhesion to plastic culture flasks (**Figure 1A**). In this study, we successfully isolated and cultured the homogeneous hDPSCs, and confirmed the adherent cells obtained in this study were human dental pulp mesenchymal stem cells, which provide an experimental basis for the next hDPSCs as ideal seed cells for disease cell therapy.

Many embryonic development-related signaling pathways, including Wnt, Nodal, BMP4 Hedgehog, FGF, Notch, and TGF-β signaling pathways (Oliverkrasinski and Stoffers, 2008; Fuming et al., 2011), regulate different aspects of pancreatic and endocrine cell development. We use Activin A, Noggin, and a combination of small molecule compounds to design and screen strategies by simulating *in vivo* embryonic pancreatic developmental pathways. The hDPSCs were induced to differentiate into definitive endoderm-like cells, pancreatic progenitor-like cells and IPCs under 2D culture conditions. After the comparation of M 1 and M 2, the results showed that we successfully established a new system suitable for inducing hDPSCs to differentiate into IPCs, which laid the foundation for subsequent research.

We physically encapsulated hDPSCs using Matrigel, a basement membrane matrix rich in laminin and collagen IV, to provide vectors and 3D culture conditions for hDPSCs. In addition, culture and induction of the differentiation of hDPSCs into insulin-secreting cells in Matrigel rich in laminin and collagen IV has not been studied.

The 3D environment not only allows the connection between the cell and the basement membrane, but also allows the cells to acquire oxygen, hormones and nutrients, as well as to remove waste. In a 3D environment of an organism, cell movement usually follows a chemical signal or molecular gradient, which is critical for the development of the organism. In 2D cultures based on “culture dishes,” cells isolated directly from higher organisms often alter their metabolism and gene expression patterns. Cells grown in a 2D environment may significantly reduce the production of specific ECM proteins and often undergo morphological changes (Gelain et al., 2006). Although 2D cell culture technology is beneficial to molecular level research, it ignores the role of tissue space microenvironment. This limits their potential to predict true biological cellular responses, leading to inconsistent outcomes in many *in vitro* and *in vivo* experiments (Pampaloni et al., 2007). In the 3D microenvironment, human embryonic stem cells differentiated into pancreatic endocrine cells with higher efficiency and more obvious response to glucose (Wang et al., 2017).

We identified IPCs obtained in Matrigel using 2D cultured IPCs as controls. The results of RT-qPCR showed that the mRNA expression levels of islet cell marker genes Pdx1, Ngn3 and Insulin in the 3D group were significantly higher than those in the 2D group. Immunofluorescence staining of frozen sections showed that cells in the cell mass were highly expressed Pdx1 and Insulin. The results indicate that 3D culture promoted the differentiation of hDPSCs into IPCs at the gene and protein levels, respectively. Glucose-stimulated insulin/C peptide release assay detects the function of IPCs in the 3D group. Glucose-stimulated insulin/C peptide release assay detects the function of IPCs in the 3D group. The 3D group IPCs responded significantly to high glucose stimulation, releasing significantly more insulin than the 2D group, and the stimulation index was about 2 times that of the 2D group. It is indicated that IPCs induced by 3D group are more sensitive and mature to glucose response. In summary, Matrigel-3D cell culture promoted the differentiation of hDPSCs into IPCs at the gene and protein levels, while significantly enhancing the release of insulin and C-peptide by IPCs.

## Data Availability Statement

The datasets generated for this study are available on request to the corresponding author.

## Ethics Statement

Written informed consent was obtained from the donors and guardians. The experiments involving human tissue were approved by the Capital Institute of Pediatrics.

## Author Contributions

BX and BN conceived and designed the experiments. BX performed the experiments, analyzed the data and drafted the manuscript. YZ and JL collected human tooth samples. BX, YZ, JL, and DF carried out isolation and culture of MSCs from human dental pulp, immunophenotype analysis by flow cytometry of MSCs. BX and ZW participated in RNA isolation, qPCR analysis, immunocytochemistry analysis and insulin and C-peptide release assay. JW, DF, XW, ZG, and BN revised the manuscript. All authors read and approved the final manuscript.

## Funding

This work was supported by the National Natural Science Foundation of China (No.81741023) and Beijing Municipal Natural Science Foundation (No.7182025).

## Conflict of Interest

The authors declare that the research was conducted in the absence of any commercial or financial relationships that could be construed as a potential conflict of interest.
